# Coexistence of recurrent chromosomal abnormalities and the Philadelphia chromosome in acute and chronic myeloid leukemias: report of five cases and review of literature

**DOI:** 10.1186/s13039-020-00501-6

**Published:** 2020-08-19

**Authors:** Jin-Ying Gong, Zhen-Hao Zhang, Wei Zhang, Hui-Jun Wang, Xiao-Fang Feng, Ji Zhou, Guo-Qing Zhu

**Affiliations:** 1 State Key Laboratory of Experimental Hematology, National Clinical Research Center for Blood Diseases, Institute of Hematology and Blood Diseases Hospital, Chinese Academy of Medical Sciences and Peking Union Medical College, 288 Nanjing Road, Heping District, Tianjin, 300020 People’s Republic of China; 2 Department of Hematology, Lymphoma Research Center, Peking University Third Hospital, 49 North Garden Road, Haidian District, Beijing, 100191 People’s Republic of China

**Keywords:** Chronic myelogenous leukemia, Acute myeloid leukemia, Balanced chromosomal abnormalities, Clonal evolution, t(9;22)

## Abstract

Progression of chronic myelogenous leukemia (CML) is frequently accompanied by cytogenetic evolution. Additional genetic abnormalities are seen in 10–20% of CML cases at the time of diagnosis, and in 60–80% of cases of advanced disease. Unbalanced chromosomal changes such as an extra copy of the Philadelphia chromosome (Ph), trisomy 8, and i(17)(q10) are common. Balanced chromosomal translocations, such as t(3;3), t(8;21), t(15;17), and inv(16) are typically found in acute myeloid leukemia, but rarely occur in CML. Translocations involving 11q23, t(8;21), and inv(16) are relatively common genetic abnormalities in acute leukemia, but are extremely rare in CML. In the literature to date, there are at least 76 Ph+ cases with t(3;21), 47 Ph+ cases with inv(16), 16 Ph+ cases with t(8;21), and 9 Ph+ cases with t(9;11). But most of what has been published is now over 30 years old, without the benefit of modern immunophenotyping to confirm diagnosis, and before the introduction of treatment regimes such as TKI. In this study, we explored the rare concomitant occurrence of coexistence current chromosomal translocation and t(9;22) in CML or acute myeloid leukemia (AML).

## Introduction

The prognostic significance of clonal evolution in CML is variable and depends on many factors, including the type of cytogenetic changes, the time and phase of emergence of clonal evolution, and other chromosomal abnormalities [1–3]. Although cytogenetic evidence of clonal evolution in CML is common as the disease progresses to accelerated or blast phase, its impact is dependent on the specific chromosomal anomalies [4]. The coexistence of t(9;22)(q34;q11.2) and recurrent chromosomal abnormalities is extremely uncommon, especially t(9;11) and t(3;21). Few such cases have been reported. To date, coexistence of t(9;11) and t(9;22) has not been reported in China. Here, we have characterized 5 cases of hematologic malignancy exhibiting coexistence of t(9;22) and recurrent chromosomal abnormalities, including two cases of t(9;11) and one case each of t(3;21), t(8;21) and inv(16).

## Materials and methods

### Patients

A retrospective study was conducted on 1382 patients with hematologic malignancies displaying t(9;22) translocations who were treated at the outpatient department or inpatient ward of the Blood Diseases Hospital (Institute of Hematology), Chinese Academy of Medical Sciences between January 2016 and December 2018. These patients included 834 males and 548 females, giving a male-to-female ratio of 3:2. Five patients (0.36%) had additional rare recurrent genetic abnormalities: 2 patients had t(9;11), 1 patient had t(3;21), 1 patient had t(8;21), and 1 patient had inv(16). The clinical and laboratory data from these 5 patients are described in detail. This study (registration no. NI2020002-EC-1) was approved by the institutional review board of the Institute of Hematology and Blood Diseases Hospital Chinese Academy of Medical Sciences and informed consent was obtained from all subjects.

### Cytogenetic analysis

Chromosomal analyses were performed by examining short-term cultures of bone marrow specimens according to standard conventional cytogenetic protocols. Fresh bone marrow was collected from each patient and cultured for 24 h (in RPMI 1640 medium, 20% calf serum) without addition of any growth factors. A methanol–glacial acetic acid fixation method was used for obtaining metaphase cells, and R-banding was performed. Analysis was performed using an Ikaros automated scanning system (Metasystems, Germany). At least 20 cells in metaphase were analyzed in each case. Karyotype descriptions are based on the International System for Human Cytogenomic Nomenclature (ISCN 2016).

### Fluorescent in situ hybridization analysis

#### FISH of interphase cells

Fluorescence in situ hybridization (FISH) was performed on interphase nuclei of samples. FISH analyses were performed to confirm Ph translocation and the presence of t(8;21). Dual color-dual fusion labeled LSI AML1/ETO and LSI BCR/ABL1 probes (Abbott Diagnostics, IL, USA), designed for their respective purposes. The slides were pretreated with 2× sodium saline citrate, pH 7.0, for 30 min at 37 °C, followed dehydrated in 70, 85, and 100% ethanol solutions for 2 min. Probes were denatured and hybridized according to the manufacturer’s instructions. Samples were rapidly washed, 10 μl of DAPI/antifade reagent (Abbott Diagnostics, IL, USA) was added to each sample, and slides were covered. Analysis was performed using an Isis system (Metasystems, Germany). Five hundred interphase cells were analyzed per specimen.

#### FISH of metaphase cells

FISH analyses of metaphase cells were performed to confirm Ph translocation and the presence of t(9;11), t(3;21), t(5;7) or inv(16). FISH analysis was performed on a R-banded slide for metaphase mapping to detect *BCR/ABL1* fusion gene and *MLL/AF9*, *EVI1*, *CBFβ*, and *EGR1/D5S721*. After karyotype analysis, a well-dispersed metaphase mitosis figure with clear band and chromosomal abnormalities was identified, photographs were taken, and coordinates were recorded. Metaphase FISH was performed on the same mitotic figure twice. Metaphase FISH was performed using a BCR/ABL1 and MLL/AF9 dual-fusion probe; an EVI1 and CBFβ dual-color separation probe, and an EGR1/D5S721 dual-color deletion probe. BCR/ABL1, CBFβ, and EGR1/D5S721 probes were from Abbott Diagnostics (Vysis, IL); MLL/AF9 and EVI1 probes were from Cytocell (Cambridge, UK). Sample slides were deparaffinized in xylene for 10 min, destained in methanol for 10 min, fixed in fresh fixative solution for 10 min, dehydrated through a graded series of ethanol solutions, and air-dried overnight. Probes were denatured and hybridized according to the manufacturer’s instructions. Since samples had been treated with a hot salt solution, the denaturation temperature was increased to 75–78 °C, and the denaturation time was extended to 5 min. Samples were rapidly washed, 10 μl of DAPI/antifade reagent was added to each sample, and slides were covered. Analysis was performed using an Isis system (Metasystems, Germany).

### Real-time quantitative RT-PCR assay

A real-time quantitative reverse transcription–polymerase chain reaction (RT-PCR) assay was performed for the detection of *BCR/ABL1,MLL/AF9,AML/ETO,CBFβ/MYH11* and *AML1-MDS/EVI1* fusion gene transcripts. RNA was extracted from bone marrow samples using Trizol reagent (Gibco-BRL, Gaithersburg, MD) according to the manufacturer’s instructions. Reverse transcription was performed on 1 μg of total RNA using random hexamers and superscript II reverse transcriptase (Gibco-BRL) as described previously. The resulting complementary DNA was subjected to PCR to amplify fusion transcripts in an ABI 7500 real-time quantitative PCR instrument. Reaction systems using primers and conditions were as described previously [5, 6]. These results were then used to calculate the copy number of each target gene and its internal reference gene *ABL1* based on a standard curve. Target gene mRNA level (%) = (copy number of target gene / copy number of *ABL1*) × 100%.

### Flow cytometric immunophenotyping

Eight-color multiparametric flow cytometry (MFC) was performed on peripheral specimens or bone marrow specimens using BD FACSCanto II instruments (BD Biosciences) according to the manufacturer’s instructions. Incubation of cells with monoclonal antibodies at 4 °C was followed by RBC lysis with NH_4_Cl for 10 min and washing with phosphate-buffered saline solution. Cells were resuspended and fixed with 1% formaldehyde. A screening panel consisting of 4 tubes was performed: (1)CD64/CD117/CD34/CD33/CD7/HLA-DR/CD38/CD45;(2)CD15/CD13/CD34/CD123/CD56/CD16/CD11b/CD45;(3)CD36/CD10/CD5/CD20/CD4/CD14/CD19/CD45;(4)TdT/MPO/CD9/CD2/cCD79a/mCD3/cCD3/CD45. Data analysis was performed using FCS Express 5 software (De Novo Software). A total of 10,000 events were acquired in each case. Major cell populations were defined by CD45/SSC (side-scatter) characteristics. All populations were further refined by forward and side-scatter gates to exclude nonspecific binding. A positive flow cytometry result was defined as the presence of circulating myeloblasts, lymphoblasts, or monocytes with aberrant antigen expression or lymphoproliferative disorder. Criteria for judging results: For each antibody, appropriate negative levels were determined by comparison with an isotype-matched control sample. Leukemia cell membrane surface antigen > 20% was judged to be positive, and intracellular antigen > 10% was judged to be positive [7].

## Results

**Fig. 1:**
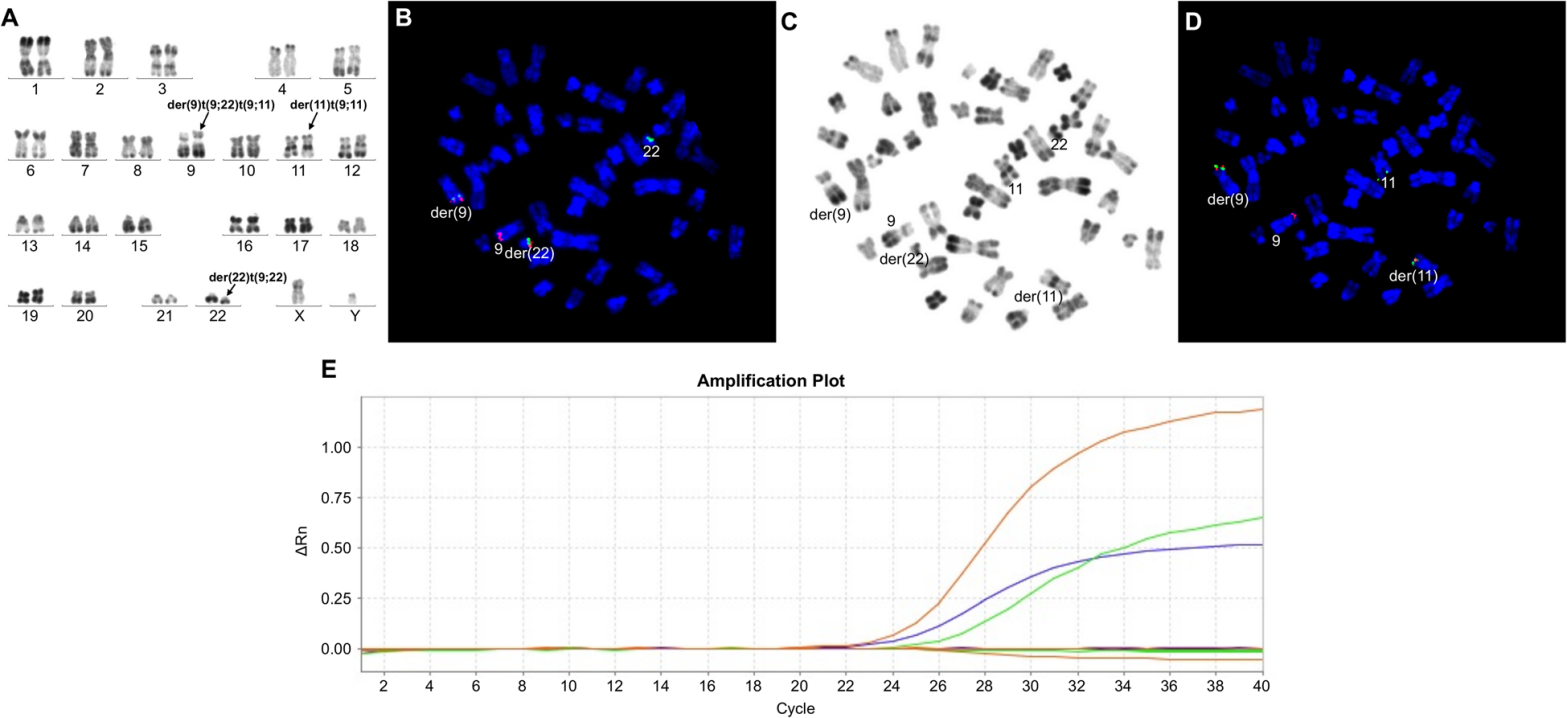
Bone marrow karyotype, metaphase-FISH, and fusion gene results of Case 1. **a** R-banded karyotype of case 1: 46,XY,der(9)t(9;22)(q34;q11.2)t(9;11)(p22;q23),der(11)t(9;11)(p22;q23),der(22)t(9;22)(q34;q11.2). Panels **b**-**d** represent FISH analyses of metaphase chromosomes corresponding to the karyotypes in panel **a**, **b** Metaphase FISH using dual color BCR-ABL1 fusion (ABL1: red; BCR: green). **c** Corresponding R-banded metaphase. **d** Sequential FISH using MLL-AF9 fusion (AF9: red; MLL: green) probe on the same metaphase cell, showing MLL-AF9 fusion. **e** BCR/ABL1 and MLL/AF9 results for patient 1. Red peak, BCR/ABL1; green peak, MLL/AF9 fusion gene transcripts; blue peak, internal reference gene *ABL1*

**Fig. 2:**
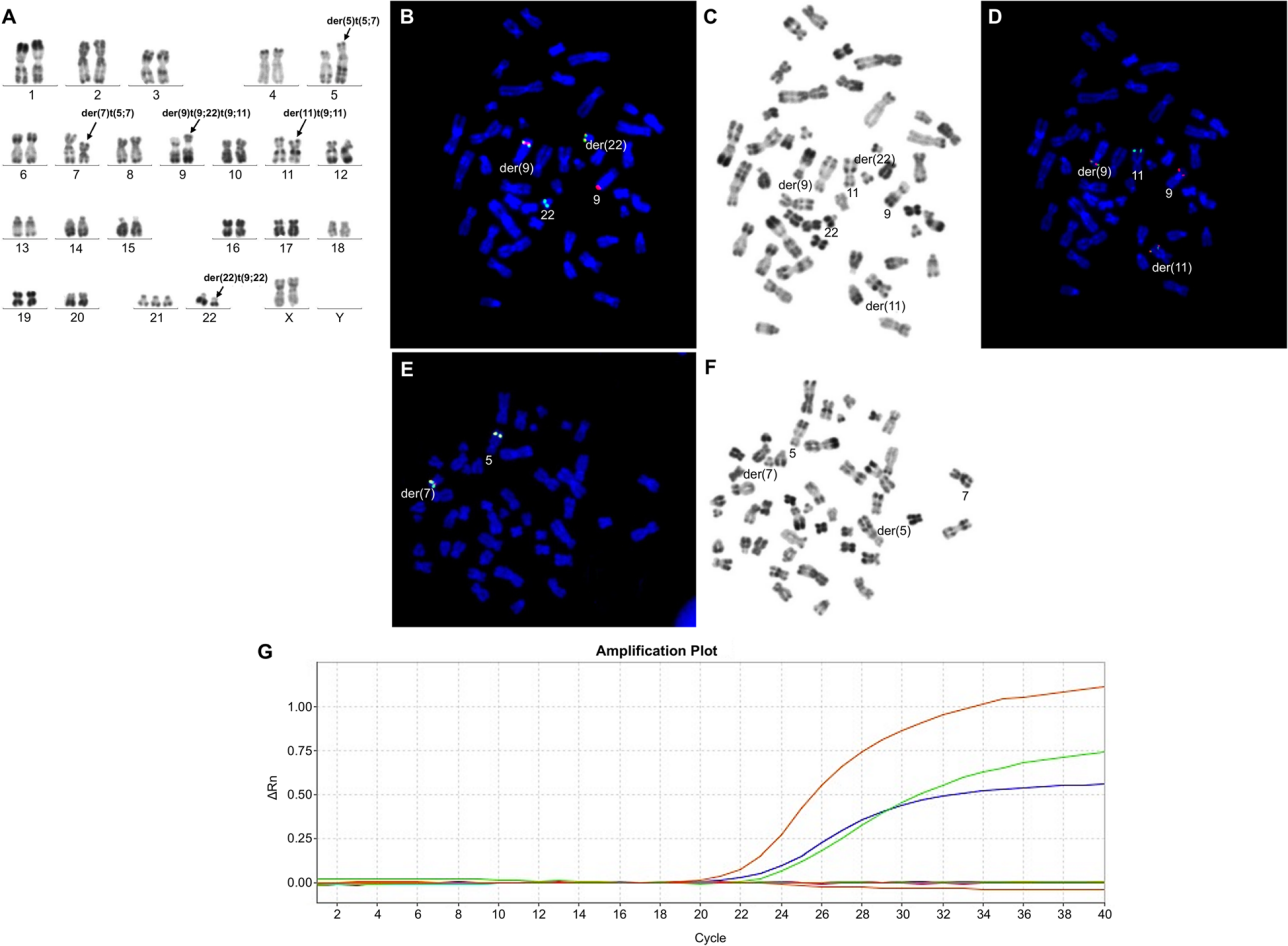
Bone marrow karyotype, metaphase-FISH, and fusion gene results of Case 2. **a** R-banded karyotype of case 2: 47,XX,t(5;7)(q31;q21),der(9)t(9;22)(q34;q11.2)t(9;11)(p22;q23),der(11)t(9;11)(p22;q23),+21,der(22)t(9;22)(q34;q11.2). Panels **b**-**d** represent FISH analysis on metaphase cells corresponding to karyotypes in panels **a** and **b** Metaphase FISH using dual color BCR-ABL1 fusion (ABL1: red; BCR: green). **c** Corresponding R-banded metaphase. **d** Sequential FISH using MLL-AF9 fusion (AF9: red; MLL: green) probe on the same metaphase cell, showing its MLL-AF9 fusion. **e** Metaphase FISH using dual color break apart probe showing PDGFRB(5q32-q33) with PDGFRB fusion (fusion red/green signal). **f** Corresponding R-banded metaphase. **g** BCR/ABL1 and MLL/AF9 results for patient 2. Red peak, BCR/ABL1; green peak, MLL/AF9; blue peak, internal reference ABL1

**Fig. 3:**
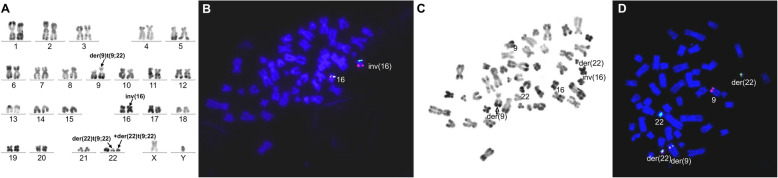
Bone marrow karyotype and metaphase-FISH results of Case 3. **a** R-banded karyotype of case 3: 47,XY,t(9;22)(q34;q11.2),inv(16)(p13;q22),+der(22)t(9;22)(q34;q11.2). Panels **b**-**d** represent FISH analysis on metaphase cells corresponding to the karyotypes in panel **a**, **b** Metaphase FISH using dual inv(16) with CBFB rearrangement (split red/green signal). **c** Corresponding R-banded metaphase. **d** FISH using BCR/ABL1 dual color fusion probe on a different metaphase cell, showing the BCR-ABL1 fusion(ABL1: red; BCR: green)

**Fig. 4:**
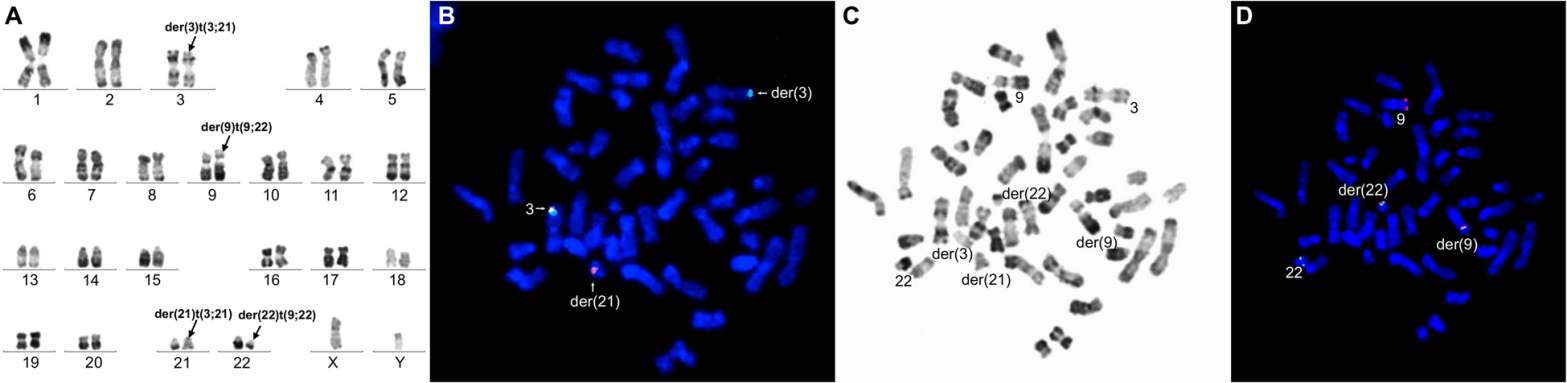
Bone marrow karyotype and metaphase-FISH results of Case 4. **a** R-banded karyotype of case 4: 46,XY,t(3;21)(q26;q22),t(9;22)(q34;q11.2). Panels **b**-**d**represent FISH analysis of metaphase cells corresponding to the karyotypes in panel **a**, **b** Metaphase FISH using dual color break apart probe EVI1 showing EVI1 rearrangement (split red/ green signal). **c** Corresponding R-banded metaphase. **d** Sequential FISH using BCR-ABL1 fusion (ABL1: red; BCR: green) probe on the same metaphase showing BCR-ABL1 fusion (ABL1: red; BCR: green)

**Fig. 5:**
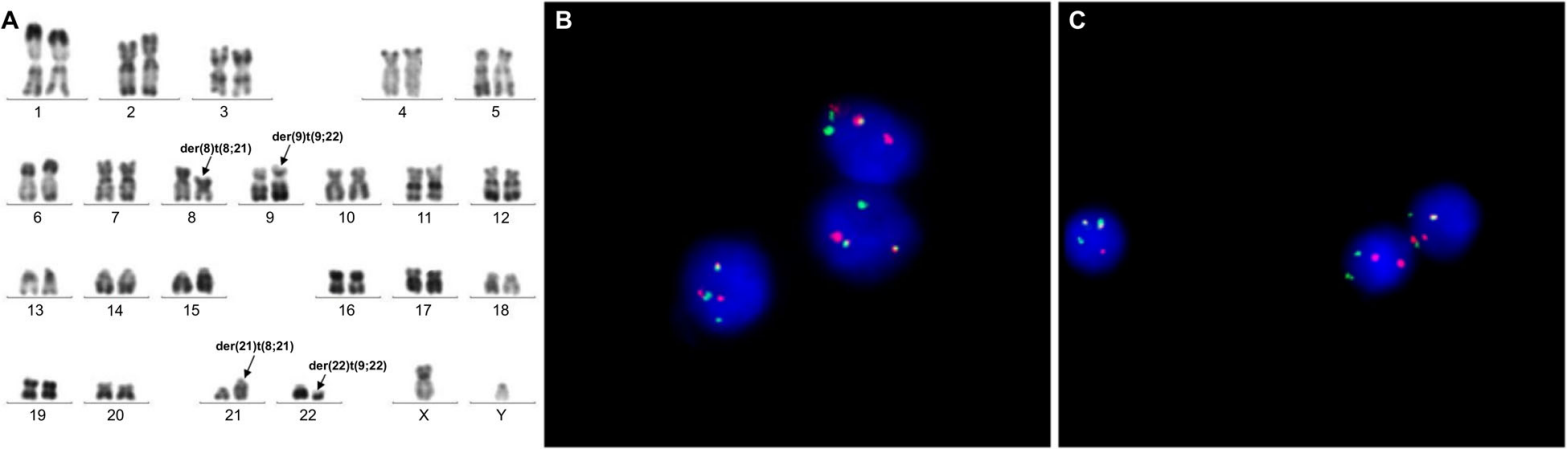
Bone marrow karyotype and interphase-FISH results of Case 5. **a** R-banded karyotype of case 5: 46,XY,t(9;22)(q34;q11.2),t(8;21)(q22;q22). Panels **b**-**c** represent FISH analysis on interphase cells corresponding to the karyotypes in panel **a**. **b**-**c** Interphase FISH using BCR/ABL1 (ABL1: red; BCR: green) dual color fusion probe, and RUNX1-RUNX1T1 (RUNX1: red; RUNX1T1: green) dual color fusion probe on different interphase cells showing a fused red-green (BCR‑ABL1) signal (yellow) and fused red-green (RUNX1-RUNX1T1) signal (yellow)

We retrospectively studied 1382 patients with hematologic malignancies accompanied by a t(9;22) translocation. Among these, 5 patients (0.36%) had additional rare recurrent genetic abnormalities in addition to t(9;22). In addition to t(9;22)(q34;q11), 2 of these 5 patients displayed the t(9;11)(p22;q23) abnormality. These 2 patients were confirmed as having both t(9;22) and t(9;11) in the same metaphase mitotic chromosome figure through karyotype analysis and BCR/ABL1 and MLL/AF9 metaphase FISH. In addition, the two abnormalities t(9;22) and t(9;11) existed in the same chromosome 9, namely der(9)t(9;22)t(9;11). Of which one case, was accompanied by t(5;7)(q31;q21) in addition to der(9)t(9;22)t(9;11). Metaphase FISH using the PDGFRB (5q32-q33) dual color break apart probe showed a PDGFRB (5q32-q33) gene on chromosome 7, confirming the t(5;7) translocation; karyotype and FISH results are shown in Figs. 1a and 2f. Quantitative PCR detection of fusion genes was also performed. The peak profile results for the *BCR/ABL1* and *MLL/AF9* fusion genes are shown in (Fig. 1e, Fig. 2g). In the other 3 patients, in addition to the t(9;22) translocation, one patient also had t(3;21)(q26;q22), one patient also had t(8;21)(q22;q22), and one patient also had inv(16)(p13;q22). Interphase FISH was performed for the patients with t(8;21). Metaphase FISH was performed for the remaining 2 patients to confirm karyotype results. Patient 3, with inv(16)(p13;q22) was tested by FISH for BCR/ABL1 and CBFβ, and gave a positive result for both genes; karyotype and metaphase FISH results are shown in Fig. 3a-d. Patient 4 with t(3;21) was positive for the *BCR/ABL1* fusion gene, and in the same mitotic figure was also positive for the EVI1 break apart probe. The breakpoint was located on chromosome 21. Molecular techniques also showed that the *BCR/ABL1* and *AML1-MDS/EVI1* fusion genes were positive; karyotype and metaphase FISH results are shown in Fig. 4a-d. Patient 5 had positive interphase FISH results for BCR/ABL1 and RUNX1-RUNX1T1. Karyotype and FISH results are shown in Fig. 5a-c. Three of the five patients were newly diagnosed with chronic myelogenous leukemia, and all relapsed after imatinib treatment. Of the 5 patients, the two relapsed patients with t(9;11) were AML-M2, and the patient with t(8;21) had accelerated phase CML. The other two cases had AML-M5 at the time of initial diagnosis, and no history of CML. The prognosis of these 5 patients was very poor, and all died within 1 week to 8 months. Their laboratory data results are shown in Table 1. The 3 patients who relapsed after imatinib treatment were all initially diagnosed with typical CML, with only the t(9;22) translocation and only the *BCR/ABL1* fusion gene abnormality present. Additional chromosomal abnormalities appeared after relapse, and the morphology and disease course also changed. The morphological and flow cytometric results of all 5 patients are shown in Table 2.

**Table 1:** Laboratory data

Case	Sex	Age	WBC	HGB	PLT	Disease stage	Clinical diagnosis	Outcome	Karyotype	RT-PCR	FISH
1	M	47	298.3	83	603	Initial diagnosis	CML	Recurrence at 9 months after CML diagnosis and imatinib treatment	46,XY,t(9;22)(q34;q11.2)[15]	BCR/ABL positive, MLL/AF9 negative	Interphase: BCR/ABL positive, MLL/AF9 undetermined
						Post-recurrence	CML blastic phase AML-M2	Died 4 months after recurrence of CML as AML-M2 and chemotherapy	46,XY,der(9)t(9;22)(q34;q11.2)t(9;11)(p22;q23),der(11)t(9;11)(p22;q23),der(22)t(9;22)(q34;q11.2)[20]	BCR/ABL positive, MLL/AF9 positive	BCR/ABL positive, MLL/AF9 positive
2	F	25	414.7	75	297	Initial diagnosis	CML	Recurrence at 1 year after CML diagnosis and imatinib treatment	46,XX,t(9;22)(q34;q11.2)[20]	BCR/ABL positive, MLL/AF9 negative	Interphase: BCR/ABL positive, MLL/AF9 undetermined
						Post-recurrence	CML blastic phase AML-M2	Died 6 months after recurrence of CML as AML-M2 and chemotherapy	47,XX,t(5;7)(q33;q21),der(9)t(9;22)(q34;q11.2)t(9;11)(p22;q23),der(11)t(9;11)(p22;q23),+21,der(22)t(9;22)(q34;q11.2)[20]	BCR/ABL positive, MLL/AF9 positive	BCR/ABL positive, MLL/AF9 positive
3	M	41	150.02	123	82	Initial diagnosis	AML-M5	Died 6 months after AML diagnosis and treatment	46,XY,t(9;22)(q34;q11.2),inv(16)(p13;q22)[17]/47,idem,+der(22)t(9;22)(q34;q11.2)[3]	BCR/ABL positive, CBFβ/MYH11 positive	BCR/ABL positive, CBFβ positive
4	M	58	46	64	34	Initial diagnosis	AML	Died 8 months after AML diagnosis and treatment	46,XY,t(3;21)(q26;q22),t(9;22)(q34;q11.2)[20]	BCR/ABL positive, AML1-MDS/EVI1 positive	BCR/ABL positive, EVI1 positive
5	M	46	113.52	88	554	Initial diagnosis	CML	Recurrence at 4 months after CML diagnosis and oral imatinib treatment (treatment voluntarily discontinued at times)	46,XY,t(9;22)(q34;q11.2)[15]	BCR/ABL positive, RUNX1-RUNX1T1 negative	Interphase: BCR/ABL positive, RUNX1-RUNX1T1 undetermined
						Post-recurrence	CML	Post-recurrence morphology and flow cytometry results indicated accelerated phase of CML. Died of internal organ bleeding 1 week after recurrence	46,XY,t(9;22)(q34;q11.2)[10]/46,idem,t(8;21)(q22;q22)[10]	BCR/ABL positive, RUNX1-RUNX1T1 positive	BCR/ABL positive, RUNX1-RUNX1T1 positive

WBC Reference range: 4.0–10.0 × 10^9^/L; HGB Reference range: Males: 120–160 g/L, Females: 110–150 g/L; PLT Reference range: 100–300 × 10^9^/L

**Table 2:** Morphology and flow cytometry results

Case	Morphology	Flow cytometry
1	Significantly active bone marrow hyperplasia. Progenitor and immature cells accounted for 82.5%. Cell bodies were non-uniform in size and round or nearly round in shape, with few azurophilic granules and visible Auer rods. Nuclei were irregular with indentations, folds, kidney shapes, and so on. Nuclear chromatin appeared as uniformly distributed fine granules, with 2–5 nucleoli.	91.81% of cells were abnormal; (+): CD33, CD13, CD117, CD123; Partial expression: HLA-DR; dim: MPO, CD64;(−): CD34, CD38, CD7, CD5, CD11b, CD56, CD19, CD20, CD10, CD4, CD14, CD36, cCD3, cCD79a
2	Significantly active bone marrow hyperplasia with increased proportion of granulocytes. The majority of cells were granulocyte progenitors, accounting for 70.5%. Cell bodies were non-uniform in size, round or nearly round in shape, had deep margins, and few azurophilic granules. Nuclei were irregular with indentations and folds. Nuclear chromatin appeared as uniformly distributed fine granules, with 2–4 nucleoli.	83.5% of cells were abnormal; (+): CD33, CD13, CD117, CD123; Partial expression: HLA-DR,CD34; dim: MPO, CD64; (−): CD38, CD7, CD5, CD11b, CD56, CD19, CD20, CD10, CD4, CD14, CD36, cCD3, cCD79a
3	Extremely active bone marrow hyperplasia. Progenitor and immature cells accounted for 84%. Cell bodies were non-uniform in size and round or nearly round in shape, with few azurophilic granules. Nuclei were irregular with indentations, folds, and so on. Nuclear chromatin appeared as uniformly distributed fine granules, with 2–4 nucleoli.	73.91% of cells were abnormal (+): CD33, CD13, CD123; Partial expression: CD34, CD117, HLA-DR dim: MPO, CD38; (−): CD7, CD5, CD11b, CD56, CD19, CD20, CD64, CD10, CD4, CD14, CD36, cCD3, cCD79a
4	Significantly active bone marrow hyperplasia. Progenitor and immature cells accounted for 46%. Cell bodies were non-uniform in size and round or nearly round in shape, and had small amounts of cytoplasm. Purple-red granules were visible in some cells. Most nuclei were distorted and folded, with 1–4 nucleoli, some of which were unclear.	28.8% of cells were abnormal; (+): CD117,HLA-DR, CD13, CD33, CD34, CD38, CD15, CD64, CD11c, MPO; (−): CD7, CD5, CD56, CD19, CD20, CD10, CD4, CD14, CD36, cCD3, cCD79a
5	Significantly active bone marrow hyperplasia with increased proportion of granulocytes. Granulocyte progenitors accounted for 4%, of which the majority were early, middle, late, and rod-shaped. Lobulated neutrophils and eosinophils were occasionally found. Basophils were not found.	Low proportion of myeloid progenitor cells on flow cytometry, accounting for 1.57% of nucleated cells. CD117 expression was lost in some cells. Abnormal expression of CD56 and CD13/CD11b differentiation antigens in some granulocytes. Eosinophils were found.

## Discussion

Chronic myelogenous leukemia progression is often accompanied by cytogenetic evolution, its impact on prognosis is dependent on the specific chromosomal anomalies that occur. The most common forms of which are translocations that produce a Philadelphia chromosome (Ph), + 8, or i(17)(q10) [8]. The coexistence of the Ph and recurrent abnormalities are extremely rare in AML and CML [9]. We describe 5 cases of a rare type of Ph+ leukemia with coexistent t(9;11), t(3;21), t(8;21) and inv(16), as assessed by cytogenetic and molecular analysis. In our study, we found that only 0.36% of patients displayed recurrent genetic abnormalities, and even fewer cases presented with t(9;22) accompanied by t(9;11) and t(3;21). To date, no cases of t(9;22) accompanied by t(9;11) have been reported in China. One case of t(9;22) accompanied by t(3;21) was reported in 2006 [10].

11q23 rearrangements in chronic myelogenous leukemia are extremely rare, accounting for less than 1% of cases reported in the literature to date [11]. CML with the t(9;22) translocation rarely incurs the additional t(9;11) during disease progression. In the last twenty years, no more than 5 cases have been reported in the literature [12–14], and none have been reported in China. Our study found that the proportion of patients with t(9;22) accompanied by the t(9;11) translocation was even lower 0.14% (2/1382). Both cases were patients initially diagnosed with CML who relapsed after imatinib treatment, and died within 6 months of relapse. When initially diagnosed with CML, the only genetic abnormality in each patient was t(9;22). The t(9;11) translocation appeared with disease progression to AML. One of these 2 patients had the t(5;7) translocation in addition to t(9;11), but the survival periods of the two patients did not differ significantly, so t(9;11) may have a greater effect on CML progression. The coexistence of t(9;22) and t(9;11) in the same clone may affect differentiation and development of pluripotent stem cells. The 11q23/MLL rearrangement may be a gene activated by pluripotent stem cells, and t(9;11) may induce loss of progenitor cells during differentiation. Therefore, the appearance of t(9;11) in cancer patients with the t(9;22) translocation enhances the malignancy of tumor cells, accelerates the disease process, and has a greater impact on prognosis.

Since 1992, a total of 5 patients worldwide have been reported to have both the t(8;21) and t(9;22) translocations at the same time. Most of these were CML patients that progressed to AML [15–18]. The fifth case was published in the Chinese Journal of Medical Genetics in 2019 [19], so it will not be described in detail here. Patient 1 had both t(9;22) and t(3;21), and patient 4 had both t(9;22) and inv(16). Both were initially diagnosed with AML-M5. Neither had a history of CML, and both died after 6–8 months of treatment. The t(3;21)(q26;q22) translocation forms the fusion gene AML1-MDS/EVI1, which can be detected by reverse transcription polymerase chain reaction (RT-PCR) or EVI1 FISH. It is common in MDS and AML [20], and very rarely appears at the same time as t(9;22)(q34;q11.2). This was the second case found in China. Liu et al [10]*.* described the case of a CML patient with t(3;21)(q26;q22) during the acceleration process. The patient in the present study had both t(3;21) and t(9;22), and had AML at the time of onset. In previous studies [21–24], t(3;21)(q26;q22) has been more common in patients with CML that progressed to AML, or patients with treatment-related MDS or AML. This additional chromosomal abnormality was considered an acquired abnormality during leukemia development and progression.

Inv(16) is more common in AML (especially the AML-M4EO subtype) where it is accompanied by an increased number of eosinophils [25, 26]. AML with Inv(16) and t(9;22) is extremely rare. Clonal evolution appearing in the process of CML acceleration or AML progression has been more frequently reported in the literature [27–32]. The case in the present study did not have a history of CML, and was initially diagnosed with AML-M5. No increase was observed in eosinophils or basophils by morphology or flow cytometry. ABL kinase mutations were negative at initial diagnosis, but positive during treatment. Patient prognosis was very poor, and the patient died after 6 months.

Tumor cell evolution and disease phenotype maybe associated with chromosomal changes. Additional chromosomal changes appearing in the context of CML are one of the most important hallmarks of disease progression. Clonal evolution in CML is a prognostic factor, and associates types of chromosome changes with disease stage and progression. For example, trisomy 8, i(17q), and complex karyotypes are genetic changes associated with poor prognosis. However, our study shows that patients with recurrent genetic abnormalities have an even worse prognosis, illustrated by 5 patients that all died within 4–9 months. Due to the extremely rare incidence and small case numbers, more cases need to be assessed in the future in to study their prognostic characteristics.

## Data Availability

All data generated or analyzed during this study are included in this published article and its additional files.

## References

[1] Zaccaria A, Testoni N, Valenti AM, Luatti S, Tonelli M, Marzocchi G (2010). Chromosome abnormalities additional to the Philadelphia chromosome at the diagnosis of chronic myelogenous leukemia: pathogenetic and prognostic implications. Cancer Genet Cytogenet.

[2] Cortes J, O’Dwyer ME (2004). Clonal evolution in chronic myelogenous leukemia. Hematol Oncol Clin North Am.

[3] Johansson B, Fioretos T, Mitelman F (2002). Cytogenetic and molecular genetic evolution of chronic myeloid leukemia. Acta Haematol.

[4] Mu Q, Ma Q, Wang Y, Chen Z, Tong X, Chen FF (2012). Cytogenetic profile of 1,863 Ph/BCR-ABL-positive chronic myelogenous leukemia patients from the Chinese population. Ann Hematol.

[5] Gabert J, Beillard E, van der Velden VH, Bi W, Grimwade D, Pallisgaard N (2003). Standardization and quality control studies of ‘real-time’ quantitative reverse transcriptase polymerase chain reaction of fusion gene transcripts for residual disease detection in leukemia – a Europe against Cancer program. Leukemia.

[6] Heesch S, Neumann M, Schwartz S, Bartram I, Schlee C, Burmeister T (2013). Acute leukemias of ambiguous lineage in adults: molecular and clinical characterization. Ann Hematol.

[7] van den Ancker W, Westers TM, de Leeuw DC, van der Veeken YFCM, Loonen A, van Beckhoven E (2013). A threshold of 10% for myeloperoxidase by flow cytometry is valid to classify acute leukemia of ambiguous and myeloid origin. Cytometry B Clin Cytom.

[8] Haferlach C, Bacher U, Schnittger S, Weiss T, Kern W, Haferlach T (2010). Similar patterns of chromosome abnormalities in CML occur in addition to the Philadelphia chromosome with or without tyrosine kinase inhibitor treatment. Leukemia.

[9] Fabarius A, Leitner A, Hochhaus A, Müller MC, Hanfstein B, Haferlach C (2011). Impact of additional cytogenetic aberrations at diagnosis on prognosis of CML: long-term observation of 1151 patients from the randomized CML study IV. Blood.

[10] Liu XP, Zhang MR, Dai Y, Zhang L, Li R, Hao Y (2006). Study of genes involved in chronic myeloid leukemia with t(3;21)(q26;q22) in blastic crisis. Zhonghua Xue Ye Xue Za Zhi.

[11] Wang W, Tang G, Cortes JE, Liu H, Ai D, Yin CC (2015). Chromosomal rearrangement involving 11q23 locus in chronic myelogenous leukemia: a rare phenomenon frequently associated with disease progression and poor prognosis. J Hematol Oncol.

[12] Suzuki K, Sugawara T, Kowata S, Utsugizawa T, Ito S, Murai K (2004). Uncommon karyotypic abnormality, t(11;19)(q23;p13.3), in a patient with blastic phase of chronic myeloid leukemia. Cancer Genet Cytogenet.

[13] Lee J, Kim DS, Lee HS, Choi SI, Cho YG (2017). A novel t(9;22;11) translocation involving 11q24 in a patient with chronic myeloid leukemia: a case report. Oncol Lett.

[14] Gutiérrez LG, Noriega MF, Laudicina A, Quatrin M, Bengió RM, Larripa I (2017). An unusual translocation, t(1;11)(q21;q23), in a case of chronic myeloid leukemia with a cryptic Philadelphia chromosome. Oncol Lett.

[15] Zhang Y, Liu Y, Liu X, An L, Huang B, Li J (2019). Co-existence of t(9;22) and t(8;21) in primary blast phase of chronic myelogenous leukemia: clinical experience and literature review. Int J Clin Exp Pathol.

[16] Yin CC, Medeiros LJ, Glassman AB, Lin P (2004). T(8;21)(q22;q22) in blast phase of chronic myelogenous leukemia. Am J Clin Pathol.

[17] Ferro MT, Steegman JL, Escribano L, Heiurichs B, Parada L, García-Sagredo JM (1992). Ph-positive chronic myeloid leukemia with t(8;21)(q22;q22) in blastic crisis. Cancer Genet Cytogenet.

[18] Najfeld V, Wisch N, Mascarenhas J, Issa L, Tripodi J, Sidhu M (2011). Development of t(8;21) and RUNX1-RUNX1T1 in the Philadelphia-positive clone of a patient with chronic myelogenous leukemia: additional evidence for multiple steps involved in disease progression. Cancer Genet.

[19] Gong J, Li J, Gai Y, Tian X, Feng X, Lin Y (2019). Co-occurrence of t(8;21)(q22;q22) and t(9;22)(q34;q11) in a case with chronic myelogenous leukemia. Zhonghua Yi Xue Yi Chuan Xue Za Zhi.

[20] Soderholm J, Kobayashi H, Mathieu C, Rowley JD, Nucifora G (1997). The leukemia-associated gene MDS1/EVI1 is a new type of GATA-binding transactivator. Leukemia.

[21] Mitani K, Ogawa S, Tanaka T, Miyoshi H, Kurokawa M, Mano H (1994). Generation of the AML1-EVI-1 fusion gene in the t(3;21)(q26;q22) causes blastic crisis in chronic myelocytic leukemia. EMBO J.

[22] Rogers HJ, Vardiman JW, Anastasi J, Raca G, Savage NM, Cherry AM (2014). Complex or monosomal karyotype and not blast percentage is associated with poor survival in acute myeloid leukemia and myelodysplastic syndrome patients with inv(3)(q21q26.2)/t(3;3)(q21;q26.2): a Bone Marrow Pathology Group study. Haematologica.

[23] Park SH, Chi HS, Cho YU, Jang S, Park CJ (2013). Evaluation of prognostic factors in patients with therapy-related acute myeloid leukemia. Blood Res.

[24] Cuenco GM, Ren R (2004). Both AML1 and EVI1 oncogenic components are required for the cooperation of AML1/MDS1/EVI1 with BCR/ABL in the induction of acute myelogenous leukemia in mice. Oncogene.

[25] Monma F, Nishii K, Shiga J, Sugahara H, Lorenzo FV, Watanabe Y (2007). Detection of the CBFB/MYH11 fusion gene in de novo acute myeloid leukemia (AML): a single-institution study of 224 Japanese AML patients. Leuk Res.

[26] Moreno-Miralles I, Pan L, Keates-Baleeiro J, Durst-Goodwin K, Yang C, Kim HG (2005). The inv(16) cooperates with ARF haploinsufficiency to induce acute myeloid leukemia. J Biol Chem.

[27] Bustamante D, Chan KR, Czuchlewski DR, Saadi AA (2012). Patterns of BCR breakpoints in patients with coexisting inv(16)(p13.1q22) and t(9;22)(q34;q11.2). Int J Hematol.

[28] Roth CG, Contis L, Gupta S, Agha M, Safyan E (2011). De novo acute myeloid leukemia with Philadelphia chromosome (BCR-ABL) and inversion 16 (CBFB-MYH11): report of two cases and review of the literature. Leuk Lymphoma.

[29] Tirado CA, Valdez F, Klesse L, Karandikar NJ, Uddin N, Arbini A (2010). Acute myeloid leukemia with inv(16) with CBFB-MYH11, 3'CBFB deletion, variant t(9;22) with BCR-ABL1, and del(7)(q22q32) in a pediatric patient: case report and literature review. Cancer Genet Cytogenet.

[30] Han E, Lee H, Kim M, Kim Y, Han K, Lee SE (2014). Characteristics of hematologic malignancies with coexisting t(9;22) and inv(16) chromosomal abnormalities. Blood Res.

[31] Merzianu M, Medeiros LJ, Cortes J, Yin C, Lin P, Jones D (2005). Inv(16)(p13q22) in chronic myelogenous leukemia in blast phase: a clinicopathologic, cytogenetic, and molecular study of five cases. Am J Clin Pathol.

[32] Wu Y, Slovak ML, Snyder DS, Arber DA (2006). Coexistence of inversion 16 and the Philadelphia chromosome in acute and chronic myeloid leukemias: report of six cases and review of literature. Am J Clin Pathol.

